# *In vitro* evaluation of dental pulp stem cells for sciatic nerve regeneration: foundations for future *in vivo* applications

**DOI:** 10.3389/fcell.2025.1528213

**Published:** 2025-02-19

**Authors:** Bruna Lopes, Ana Catarina Sousa, Patrícia Sousa, Alícia de Sousa Moreira, André Filipe Coelho, Luís Atayde, António J. Salgado, Stefano Geuna, Rui Alvites, Ana Colette Maurício

**Affiliations:** ^1^ Departamento de Clínicas Veterinárias, Instituto de Ciências Biomédicas de Abel Salazar (ICBAS), Universidade do Porto (UP), Porto, Portugal; ^2^ Centro de Estudos de Ciência Animal (CECA), Instituto de Ciências, Tecnologias e Agroambiente da Universidade do Porto (ICETA), Porto, Portugal; ^3^ Associate Laboratory for Animal and Veterinary Science (AL4AnimalS), Lisboa, Portugal; ^4^ Campus Agrário de Vairão, Centro Clínico de Equinos de Vairão (CCEV), Vairão, Portugal; ^5^ Life and Health Sciences Research Institute (ICVS), School of Medicine, University of Minho, Campus de Gualtar, Braga, Portugal; ^6^ ICVS/3B's Associate Lab, PT Government Associate Laboratory, Braga/Guimarães, Portugal; ^7^ Department of Clinical and Biological Sciences, Cavalieri Ottolenghi Neuroscience Institute, University of Turin, Turin, Italy; ^8^ Instituto Universitário de Ciências da Saúde (IUCS) - Cooperativa de Ensino Superior Politécnico e Universitário (CESPU), Gandra, Portugal

**Keywords:** biomaterials, conditioned media, human dental pulp stem cells, peripheral nerve injury, regenerative medicine

## Abstract

**Introduction:**

Peripheral nerve injuries, resulting from trauma or medical interventions, present significant clinical challenges due to their severe physiological and functional impacts. Despite various therapeutic approaches, optimal methods for promoting nerve regeneration remain difficult to obtain. This study is a preliminary step towards the future use of chitosan nerve guide conduits combined with human dental pulp stem cells and their conditioned media to promote nerve regrowth in a rat model with severe sciatic nerve damage.

**Methods:**

Preliminary characterization of conditioned medium from human dental pulp stem cells identified key regenerative biomarkers using a Multiplexing LASER Bead analysis. The human dental pulp stem cells’ cytocompatibility with Reaxon® chitosan biomaterial was confirmed through viability and metabolic assays in a PrestoBlue assay, along with scanning electron microscopy and energy-dispersive X-ray spectroscopy analyses.

**Results:**

These *in vitro* assessments validated the therapeutic potential of the combinations for nerve regeneration.

**Discussion:**

Future *in vivo* experiments will involve applying these combinations in a rat model, with functional assessments to evaluate efficacy. This research aims to establish human dental pulp stem cells and nerve guide conduits as viable treatments for peripheral nerve injury, offering promising directions for clinical applications.

## 1 Introduction

Peripheral nerves are distributed throughout the body, often superficially, and are particularly susceptible to damage, making nerve injuries common (Lam and Leung, 2024; Ronchi et al., 2023). These injuries are associated with poor functional outcomes, significantly impairing physical and physiological performance by causing sensory and motor function loss (Lopes et al., 2022). Additionally, peripheral nerve injuries (PNI) cause sensory and motor deficits, with significant clinical and economic impacts (Ronchi et al., 2023). Defects smaller than 4 cm generally have a favorable prognosis, with regeneration occurring at a slow rate of about 1–3 mm per day, depending on the injury’s location (proximal or distal) (Dogny et al., 2024). The time interval between the injury and nerve regeneration is crucial in determining the success of functional recovery and preventing muscle atrophy (Escobar et al., 2023). Defects larger than 4 cm do not regenerate effectively resulting in worse outcomes, and in these cases the gold standard for repairing these lesions is the autologous nerve grafting (Crabtree et al., 2024). However, this approach is invasive and requires harvesting nerves from a healthy secondary site, which can result in sensory loss, scarring and neuroma formation (Lopes et al., 2022; Escobar et al., 2023). Alternative treatments for PNI are needed to address limitations and improve patients’ quality of life.

While peripheral nerves possess an intrinsic ability to regenerate, the process is unpredictable and depends on the injury’s severity and type. Full sensory and functional recovery remains a significant challenge, motivating researchers to explore novel therapeutic strategies (Lam and Leung, 2024; Lopes et al., 2022; Angius et al., 2012; Alvites et al., 2018).

In recent years, numerous studies have developed new techniques for promoting axonal regeneration while preserving healthy, functioning nerves, with the goal of enhancing recovery from PNI (Dogny et al., 2024; Braga Silva et al., 2021; Lischer et al., 2023). Nerve guide conduits (NGCs) are a viable alternative solution for nerve repair, without sacrificing other healthy functioning nerves (Lischer et al., 2023). NGC are useful to bridge the severed ends of a nerve, improving and offering better conditions for nerve regeneration, while also preventing interference from the surrounding tissue (Sun et al., 2024). For that reason, the use of mesenchymal stem cells (MSCs) in combination with NGCs is one of the possibilities for an ideal treatment. MSCs offer several advantages, such as their accessibility, adherence to plastic surfaces, multipotency, ability to proliferate and differentiate, in the presence of adequate differentiation media, into various cell types (adipocytes, osteoblasts, chondrocytes and neuroglial cells) (Stefanska et al., 2024; Pittenger et al., 2019; Voga et al., 2020). These cells can promote regeneration through cell differentiation into tissue-specific cell types, cell-to-cell contact and release of neurotrophic factors (Lopes et al., 2022; Candelise et al., 2023). For example, under the right conditions, they can differentiate into Schwann cell-like cells, which are beneficial for peripheral nerve regeneration (Sharifi et al., 2024). They are known for their capacity to proliferate effectively and retain multi-lineage potential over extended periods (Stefanska et al., 2024; Uzunlu and Ogurtan, 2024).

In peripheral nerve regeneration, MSCs have an important role due to their ability to release and produce a diverse array of biochemical and molecular factors, denominated as conditioned medium, that enhances axonal growth. This recent topic of research has some advantages over the use of cell-based applications. MSCs secrete a variety of growth factors, including nerve growth factor (NGF), brain-derived neurotrophic factor (BDNF), neurotrophin-3 (NT-3), glial cell-derived neurotrophic factor (GDNF), insulin-like growth factor 1 (IGF-1), vascular endothelial growth factor (VEGF), epidermal growth factor (EGF), basic fibroblast growth factor (bFGF), transforming growth factor-beta (TGF-β), and platelet-derived growth factor (PDGF), all of which are crucial for axonal growth and extension (Lischer et al., 2023; Salgado et al., 2010). MSCs also enhance nerve regeneration through paracrine effects on endogenous peripheral glial cells (Sharifi et al., 2024). Additionally, the potential risks of MSC-based therapies, including thromboembolism, fibrosis and uncontrolled proliferation, increased the interest in investigating the therapeutic potential of conditioned medium (CM) as an alternative therapeutic option (Mohd Nor et al., 2023; Younes et al., 2023).

Human dental pulp stem cells (hDPSCs) are a type of MSCs with typical stem cells’ characteristics like plasticity, high proliferation, self-renewal, and multi-lineage differentiation (Stefanska et al., 2024; Fujii et al., 2023). Additionally, studies have shown that hDPSCs can also differentiate into non-mesodermal tissues, such as neurons (Mattei and Delle Monache, 2023). Research has highlighted the regenerative potential of hDPSCs, particularly through the secretion of various bioactive molecules (Younes et al., 2023). Conditioned medium from hDPSCs (hDPSCs-CM) contains numerous bioactive factors, including anti-inflammatory cytokines, interleukins (IL-10, IL-13), follistatin, transforming growth factor-beta 1 (TGF-β1), hepatocyte growth factor, and neural cell adhesion molecule-1. These cytokines help reduce inflammation, promote progenitor cell proliferation, and improve tissue repair (Mattei and Delle Monache, 2023; Yamada et al., 2019; Li et al., 2024). Recent studies on hDPSCs suggest that they may facilitate *in vivo* nerve regeneration by supporting the differentiation of endogenous nerve cells (Luo et al., 2021). Uzunlu *et al.*, studied the effects of hDPSCs on motor function recovery. The morphological characteristics of the sciatic nerve were evaluated in rats following a crush injury using histological analyses and functional assessments to determine the extent of regeneration (Uzunlu and Ogurtan, 2024). The results demonstrated that hDPSCs significantly improved motor function, as indicated by increased sciatic functional index values, and enhanced nerve regeneration with preserved perineurium structure and reduced edema in the treated groups compared to the controls (Uzunlu and Ogurtan, 2024). Luo et al. (2021), investigated the use of a bioactive hydrogel-based nerve conduit for the repair of a 15-mm sciatic nerve gap in rats. The hDPSCs were used because of their ability to differentiate into neuron and Schwann-like cells, as well as their secretion of neurotrophic factors that facilitate nerve regeneration. A study demonstrated that the combination of DPSCs and NGCs effectively promoted both nerve tissue regeneration and functional recovery, with results comparable to those of traditional nerve grafts (Luo et al., 2021). For these reasons, hDPSCs are regarded as promising possibilities for stem cell-based therapies for PNI (Luo et al., 2018). Furthermore, the significant role of hDPSCs-CM in promoting neuroprotection and neurogenesis has been well-documented in numerous *in vitro* and *in vivo* studies (Mohd Nor et al., 2023; Kichenbrand et al., 2019; Martins et al., 2017). Barone et al., demonstrated that hDPSCs-derived soluble factors, combined with a nanostructured scaffold, support vascular network formation *in vivo*, highlighting their potential in enhancing tissue repair through improved vascularization (Barone et al., 2023). Casos-Perera et al., used rat models to demonstrate that hDPSCs-CM enhances tissue repair, assessed through histological analysis and functional tests. These results showed improved regeneration and recovery attributed to growth factors and cytokines that support cell survival and reduce inflammation (Cases-Perera et al., 2022). Similarly Zhou et al. (2021), demonstrated in *in vivo* rat models that hDPSCs-CM enhances stem cell priming. Their results, obtained through histological assessments and functional evaluations, showed increased tissue regeneration and improved cellular activity in the treated groups compared to controls (Zhou et al., 2021). Building on these findings, this study provides a detailed characterization of the biomolecular profile of hDPSCs-CM and its functional relevance to peripheral nerve regeneration. By identifying specific biomarkers and their roles in modulating immune responses, enhancing vascularization, and promoting axonal growth, our data complement existing research by elucidating the underlying mechanisms that make hDPSCs-CM a potent therapeutic agent. In addition, hDPSCs have been extensively studied in combination with scaffolds to enhance peripheral nerve regeneration. By integrating hDPSCs into NGC and other biomaterials, researchers have observed significant improvements in axonal regrowth, reduction of inflammatory responses, and restoration of motor function. These scaffolds provide structural support while leveraging the neuroprotective and regenerative capabilities of hDPSCs. Furthermore, hDPSCs have shown significant promise when combined with various scaffolds, such as hydrogels and chitosan conduits, to facilitate nerve regeneration. For example, Luo et al., reported that hDPSCs integrated into a bioactive hydrogel-based nerve conduit successfully repaired a 15-mm sciatic nerve gap in rats, promoting both functional recovery and nerve tissue regeneration (Luo et al., 2021). Uzunlu and Ogurtan (2024), demonstrated that hDPSCs significantly improved motor function recovery in a sciatic nerve crush injury model, as evidenced by enhanced nerve regeneration, reduced edema, and preserved perineurium structure. These findings highlight the therapeutic potential of hDPSCs, particularly when integrated with supportive biomaterials, offering a promising approach for addressing the challenges of PNI.

The research process begins with *in vitro* studies, which validate the therapeutic combinations to use *in vivo*. For that reason, in researches focused in PNI, *in vitro* testing is normally followed by animal model testing, with the rat sciatic nerve being a commonly used animal model due to its accessibility, appropriate size, and ease of monitoring functional recovery (Lopes et al., 2023).

The aim of this article is to establish the necessary groundwork to proceed to *in vivo* testing. For this reason, the viability and adherence of hDPSCs when cultured in the presence of the selected NGC (Reaxon^®^) had to be confirmed to ensure that the cells remained viable. Since the CM is a safer alternative for harnessing the benefits of hDPSCs, two passages (P4 and P7) were studied to determine which one would be more effective for use in therapeutic groups during the *in vivo* experiment. The analysis focused on identifying differences in the CM profile between the passages, considering factors such as anti-inflammatory properties, immune recruitment, and healing potential. The novelty of this work is in evaluating the adherence and viability of hDPSCs when in contact with Reaxon^®^ and exploring the therapeutic potential of their CM across passages, aiming to develop safer treatments for PNI.

## 2 Materials and methods

### 2.1 Preparation of hDPSC and hDPSC conditioned medium

The hDPSCs did not require isolation as they are widely used, well studied, and their positive therapeutic outcomes are well documented. Previously our group extensively characterized these cells, confirming their nature as MSCs (Machado et al., 2023; Caseiro et al., 2018; Sousa et al., 2024; Sousa et al., 2022). For this reason, a commercially available cell line was used, hDPSCs from AllCells, LLC, Alameda, CA, United States (Cat. DP0037F, Lot no. DPSC090411–01). The commercial hDPSC cell line used in this study is a primary cell line directly derived from human dental pulp stem cells. It has been extensively characterized by the supplier, confirming essential primary cell properties. The cells were then expanded and cultured under standard conditions (37°C, 5% CO_2_, and a humidified atmosphere) using a basal medium composed of αMEM, GlutaMAXTM Supplement, no nucleosides (Gibco, 32,561,029), which was supplemented with 10% (*v*/v) fetal bovine serum (FBS) (Gibco, A3160802), 100 IU/mL penicillin, 0.1 mg/mL streptomycin (Gibco, 15,140,122), 2.05 μg/mL amphotericin B (Gibco, 15,290,026), and 10 mM HEPES solution (Gibco, 15,630,122). For cryopreservation, cells were stored in the basal medium supplemented with 10% dimethyl sulfoxide (DMSO) (Sigma-Aldrich^®^) in cryovials containing at least 1 × 10^6^ cells. Prior to any assay, the cells were thawed in a 37°C water bath, collected, centrifuged, resuspended in the basal medium, counted, cultured, and maintained under the previously mentioned standard conditions. For each experimental assay, the culture medium was discarded, cells were rinsed with PBS and then detached using 0.25% Trypsin-EDTA (Sigma-Aldrich^®^) through a 3-min incubation under standard conditions. Following centrifugation (1,600 rpm, 10 min) and supernatant removal, cell count, and viability were assessed using a Trypan blue exclusion assay (Invitrogen™) and an automatic cell counter (Countess II FL Automated Cell Counter, Thermo Fisher Scientific^®^).

Once the cell cultures reached approximately 70%–80% confluence, the medium was removed, and the culture flasks were gently washed two to three times with Dulbecco’s phosphate-buffered saline (DPBS). Following this, the flasks were washed again two to three times with the basal medium composed of αMEM, without any additional supplements. For the conditioning process, unsupplemented DMEM/F12 GlutaMAXTM (10565018, Gibco^®^, Thermo Fisher Scientific^®^, Waltham, MA, United States) was added to the flasks, which were then incubated under standard conditions. The CM, rich in factors secreted by the cells, was collected after 48 h of conditioning. The collected hDPSCs-CM was concentrated five times (5×). After collection, the medium was centrifuged for 10 min at 1,600 rpm, the supernatant was collected, and then filtered using a 0.2 μm syringe filter (Filtropur S, PES, Sarstedt^®^, Nümbrecht, Germany). For the concentration process, Pierce™ Protein Concentrator, 3k MWCO, 5–20 mL tubes (88525, Thermo Scientific^®^, Waltham, MA, United States) were used. Before use, the concentrators were sterilized according to the manufacturer’s instructions. Briefly, the upper chamber of each concentrator was filled with 70% ethanol (v/v) and centrifuged at 300 g for 10 min. After centrifugation, the ethanol was discarded, and the same process was repeated with DPBS. Each concentrator was subjected to two cycles of this procedure, followed by a 10-min drying period in a laminar flow hood. Finally, the upper chambers were filled with the initial CM (at 1× concentration) and underwent another round of centrifugation, repeated as needed to achieve the desired 5× concentration. The concentrated CM was stored at −20°C and later analyzed using the Luminex™ 200 system (Luminex, Austin, TX, United States) by Eve Technologies Corp. (Calgary, Alberta) to identify specific biomarkers.

### 2.2 Analysis of hDPSCs conditioned medium

To identify specific chemokines and growth factors produced and secreted by hDPSCs, the CM was analyzed. The hDPSCs at an earlier passage (P4) *versus* a later one (P7) were used for the conditioning process and to compare both passages. This study used Luminex xMAP technology for multiplexed quantification of 48 Human cytokines, chemokines, and growth factors. Forty-eight markers were simultaneously measured in the samples using Eve Technologies’ Human Cytokine Panel A 48-Plex Discovery Assay^®^ (MilliporeSigma, Burlington, Massachusetts, United States) according to the manufacturer’s protocol. The 48-plex consisted of sCD40L, Epidermal Growth Factor **(EGF)**, Eotaxin, FGF-2, FLT-3 Ligand, Fractalkine, Granulocyte Colony Stimulating Factor **(G-CSF)**, Granulocyte-Macrophage Colony Stimulating Factor **(GM-CSF)**, Human Growth Regulated oncogene/Keratinocyte Chemoattractant **(GROα)**, IFN-α2, Interferon Gama **(IFN-γ)**, Interleukins (**IL**-1α, IL-1β, IL-1RA, IL-2, IL-3, IL-4, IL-5, IL-6, IL-7, IL-8, IL-9, IL-10, IL-12 (p40), IL-12 (p70), IL-13, IL-15, IL-17A, IL-17E/IL-25, IL-17F, IL-18, IL-22, IL-27), IP-10, Monocyte Chemoattractant Proteini-1 **(MCP-1)**, MCP-3, M-CSF, Macrophage derived Chemokine **(MDC)**, MIG/CXCL9, Macrophage Inflammatory Protein **(MIP**), MIP-1α, MIP-1β, Palete-derived Growth Factor **(PDGF-AA)**, PDGF-AB/BB, Regulated on Activation Normal T Cell Expressed and Secreted **(RANTES)**, Transforming Growth Factors **(TGFα)**, Tumor Necrosis Factor **(TNF-α)**, TNF-β, and Vascular Endothelial Growth Factor **(VEGF-A)**. The function of each biomarker is grouped in Table 1, along with its relevance in the study of peripheral nerve regeneration. Assay sensitivities of these markers range from 0.14–50.78 pg/mL for the 48-plex.

**TABLE 1:** Biomarkers grouped by functional category, with their primary functions and relevance to peripheral nerve regeneration.

Category	Biomarkers	Primary function	Relevance for peripheral nerve regeneration
Anti-Inflammatory	IL-10, IL-1RA, TGFα, TGFβ	Reduces inflammation	Creates a supportive environment for nerve repair by lowering chronic inflammation
Pro-Inflammatory	IL-1, IL-6, TNF, IFN	Initial inflammation response	Prepares nerve tissue for repair by aiding in clearing cellular debris
Immune Modulators	Interleukins (IL), G-CSF, GM-CSF, M-CSF	Immune coordination and activation	Regulate immune response and promote cellular support needed for nerve tissue repair
Pro-Vascular	VEGF-A, PDGF, EGF, FGF-2	Blood vessel formation and cell nutrition	Provides nutrients and oxygen to damaged tissue, crucial for supporting regeneration
Chemotaxis	Eotaxin, MCP, GRO, MIG/CXCL9, RANTES, MIP	Attracts supportive cells	Brings cells to injury site, preparing the area for repair and recovery
Neural Growth	FLT-3 Ligand, Fractalkine	Stimulates growth and axon guidance	Facilitates regeneration and reconnection of damaged nerve fibers

### 2.3 Cytocompatibility assessment

For further *in vivo* studies with Reaxon^®^ NGCs, the compatibility with hDPSCs was evaluated using a PrestoBlue™ viability assay. Presto Blue™ from Invitrogen (A13262; Thermo Scientific, Waltham, United States) is a ready-to-use reagent for analysis of live cells. Living cells reduce the reagent to resazurin, which change color from blue to red altering the solution fluorescence. This conversion makes the assay measurable in absorbance as an indication of cell viability and allows a quantitative evaluation of cell proliferation. As a result, cell viability and proliferation were evaluated by measuring absorbance at a mean of 570 nm, reflecting the metabolic activity of the cells. Four experimental groups were defined: (1) hDPSCs cultured in direct contact with Reaxon^®^ NGC in basal medium, (2) hDPSCs cultured in indirectly contact with Reaxon^®^ in basal medium, (3) hDPSCs cultured in basal medium as a negative control, and (4) hDPSCs cultured in basal medium with 10% DMSO instead of 10% FBS as a positive control. In the direct contact method, hDPSCs were seeded directly onto the inner surface of the Reaxon^®^ NGCs, allowing immediate interaction between the cells and the scaffold. In contrast, the indirect contact method involved placing the Reaxon^®^ NGC within the well of a multi-well plate containing a suspension of hDPSCs. This setup evaluated the influence of the NGC’s proximity on cellular behavior without direct adhesion to its surface. Non-treated plates were utilized in the direct contact method to prevent cell adherence to the well bottom, ensuring that cellular growth was restricted to the NGC surface. Additionally, hDPSCs were seeded at a density of 6,000 cells/cm^2^ in a volume of 500 μL of basal medium per well. This volume was selected to ensure uniform cell distribution across the well and to maintain optimal culture conditions. Regarding the analysis of metabolic activity using the PrestoBlue™ assay, this method measured overall metabolic activity within the well, including cells adherent to the NGC and any that may have settled elsewhere. The use of non-treated plates in the direct contact method minimized cell adherence to the bottom of the well, ensuring that observed cell growth and metabolic activity primarily reflected cells on the NGC surface. In the first and second group, Reaxon^®^ NGCs were cut to appropriate lengths, to fit within the well length placed in a row of wells in a 24-well plate. Metabolic activity was measured at 24, 72, 120, 168, and 216 h, in quadruplicate for each group at each time point. At each evaluation moment, the medium was replaced with fresh complete medium containing 10% (v/v) of PrestoBlue™ cell viability reagent (Invitrogen, A13262). Unseeded wells served as blanks. To assess this, plates were incubated under standard conditions for 1 hour. After incubation, the supernatant from each well was transferred to a 96-well plate, and absorbance was measured at both 570 nm and 595 nm using a Thermo Scientific™ Multiskan™ FC microplate photometer. The corrected absorbance values were obtained by subtracting the 595 nm reading from the 570 nm reading for each well, providing normalized values. After removing the PrestoBlue™ residue with PBS, fresh medium was added, and the plates were maintained under standard conditions for further assessments. Data were then processed and normalized to the control group, with results presented as a percentage of viability inhibition compared to the controls.

### 2.4 Scanning electron microscopy

Following the cytocompatibility assessment of hDPSCs with Reaxon^®^, a scanning electron microscopy (SEM) analysis and energy-dispersive X-ray spectroscopy (EDS) was conducted. This analysis utilized a high-resolution (Schottky) Environmental Scanning Electron Microscope equipped with X-ray Microanalysis and Electron backscattered diffraction (FEI Quanta 400 FEG ESEM/EDAX Genesis X4M). The microscope operated in high vacuum mode at an acceleration mode of 15 kV SEM. Reaxon^®^ NGCs were cut transversely to fit the diameter of a well in a 24-well plate and then longitudinally. Each half-NGC was placed inside a well, and hDPSCs were seeded at a density of 6,000 cells/cm^2^ with basal culture medium on the internal and external surface of the cut NGCs. The cells were cultured for 216 h with medium changes every 2–3 days. Following this period, wells were washed three times with 0.1 M HEPES buffer (Merck^®^, PHG0001). Cells on the NGCs’ inner surface was fixed with 2% buffered glutaraldehyde (Merck^®^, G7651) and left overnight. After, cells were washed in three cycles of 5 minutes with 0.1 M HEPES buffer with gentle agitation. Samples were then dehydrated through a graded ethanol series (50%, 70%, 90%, 99%), with each concentration applied 2–3 times for 10–15 min. At last, samples were infiltrated with a graded series of hexamethyldisilazane (HMDS) (Merck^®^, 440191) in ethanol for 15 min and incubated with HMDS alone for another 15 min. After removing HMDS, plates were left overnight in a laminar flow chamber for complete evaporation. Prior to SEM and EDS analysis, samples were coated with gold/palladium for 80 s using a 15 mA current.

### 2.5 Neurogenic differentiation assay

For neurogenic differentiation, 4 × 10^3^ cells/cm^2^ hDPSCs at P7 were seeded into a 12-well plate. The plate was maintained under standard conditions until cells reached 70%–80% confluency. Media were removed from all the wells and the neurogenic differentiation medium (MSC Differentiation Medium, PromoCell^®^) was allocated in 8 wells, the other 2 wells were used as control and maintained with the usual culture medium. Cells were maintained under differentiation for 4 days, and media changed every 48 h. Previously our group extensively characterized these cells, confirming their nature as MSCs.

### 2.6 Statistical analysis

Statistical analysis was carried out using GraphPad Prism version 6.00 for Mac OS x (GraphPad Software, La Jolla, California, United States). Data were expressed as mean ± SEM when appropriate. Group comparisons were conducted using parametric tests. A value of P < 0.05 is considered statistically significant. Significance of the results is showed according to P values by the symbol (∗), (∗) corresponding to 0.01 ≤ P < 0.05, (∗∗) to 0.001 ≤ P < 0.01, (∗∗∗) to 0.0001 ≤ P < 0.001 and (∗∗∗∗) to P < 0.0001.

## 3 Results

### 3.1 Analysis of hDPSCs conditioned medium

**FIGURE 1:**
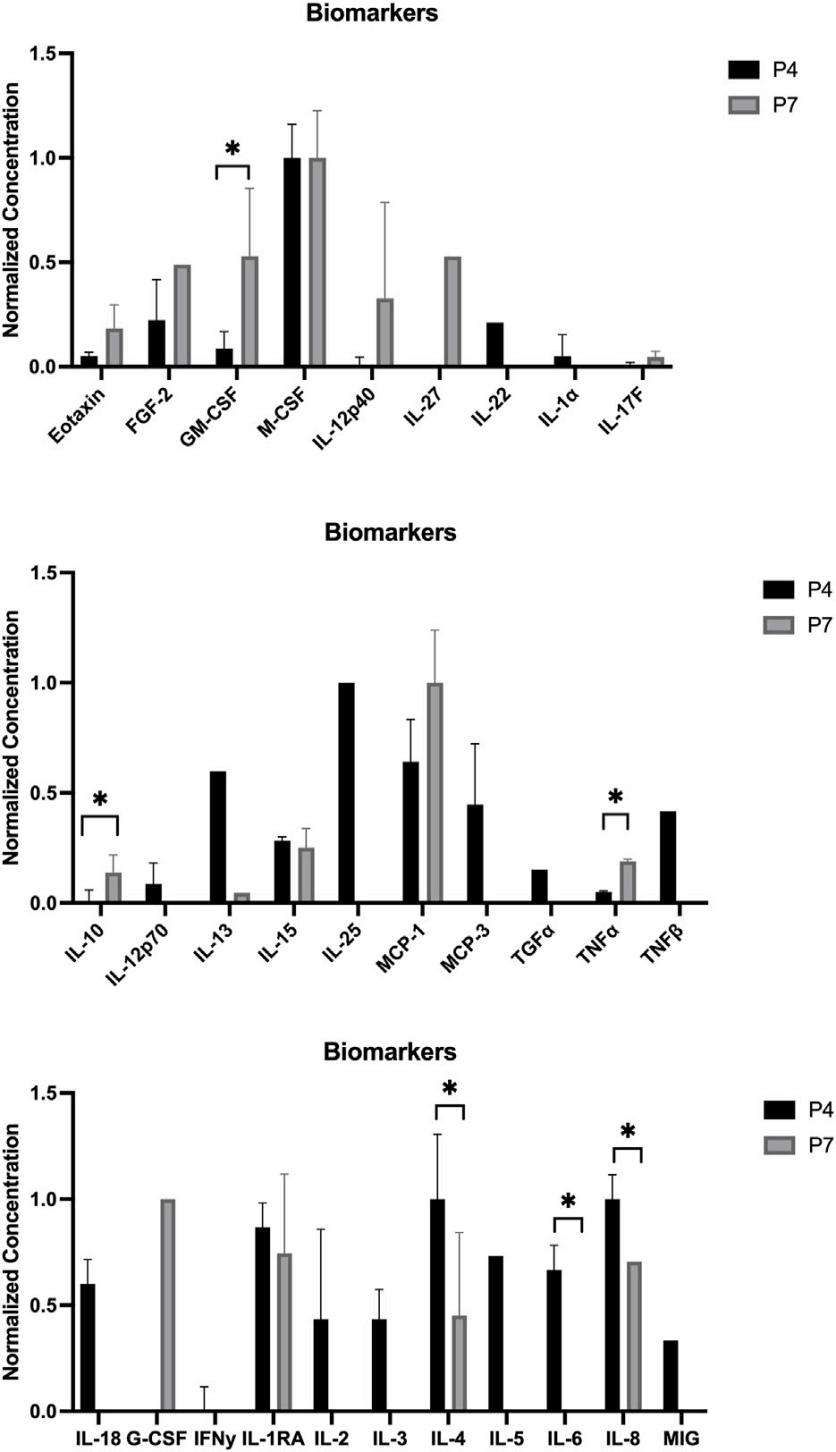
Normalized concentration of cytokines in the hDPSCs-CM in P4 and P7 (mean ± SEM): Top: Eotaxin, FGF-2, GM-CSF (p = 0.484), M-CSF, IL-12p40, IL-27, IL-22, IL-1a, and IL-17F. Middle: IL-10 (p = 0.473), IL-12p70, IL-13, IL-15, IL-25, MCP-1, MCP-3, TGFα, TNFα, and TNFβ. Bottom: IL-18, G-CSF, IFNγ, IL-1RA, IL-2, IL-3, IL-4 (p = 0.128), IL-5, IL-6 (p = 0.377), IL-8, and MIG. Results significances are presented through the symbol (*), according to the p-value, with one, two, three, or four symbols, corresponding to 0.01 < *p* ≤ 0.05; 0.001 < *p* ≤ 0.01; 0.0001 < *p* ≤ 0.001 and *p* ≤ 0.0001.

The outcomes of the hDPSCs-CM analysis are displayed in Figure 1. The average concentration of each biomarker identified in the hDPSCs-CM samples can be found in Table 2. In the hDPSCs-CM, 30 biomarkers were detected: Eotaxin, FGF-2, GM-CSF, M-CSF, IL-12p40, IL-27, IL-22, IL-1a, IL-17F, IL-10, IL-12p70, IL-13, IL-15, IL-25, MCP-1, MCP-3, TGFα, TNFα, TNFβ, IL-18, G-CSF, IFNγ, IL-1RA, IL-2, IL-3, IL-4, IL-5, IL-6, IL-8 and MIG. Most biomarkers were detected in both P4 and P7, although differences in their concentrations were observed. When biomarkers were only present in one passage, they were more frequently found in P4. For instance, P4 exhibited a higher presence of cytokines such as IL-15, IL-25, MCP-3, and IL-6 compared to P7. However, in specific cases such as GM-CSF and MCP-1, P7 showed higher concentrations, indicating that a later passage might be more favorable for certain biological functions, such as promoting immune response and chemotaxis. At 48 h, P4 displayed higher levels of anti-inflammatory factors like IL-4 and IL-5, alongside growth factors such as G-CSF and IFNγ, highlighting its potential for tissue repair. Although both passages show valuable biomarker profiles, P4 generally displays a greater variety and higher concentrations of important bioactive molecules. This suggests that P4 may offer more comprehensive support for therapeutic applications requiring a wide range of biological activities for optimal tissue regeneration.

**TABLE 2:** The hDPSCs-CM analysis with mean normalized concentration values for each biomolecule in P4 and P7 (mean ± SEM).

Biomolecule	Mean ± SEM (P4)	Mean ± SEM (P7)	Statistical differences
Eotaxin	1.98 ± 0.45	1.40 ± 0.79	nd
FGF-2	6.41 ± 5.02	3.51 ± 0.00	nd
FLT-3L	0.19 ± 0.03	0,17 ± 0.02	nd
GM-CSF	2.86 ± 2.14	3.79 ± 2.25	*
G-CSF	0.00 ± 0.00	0.18 ± 0.00	nd
IFNγ	0.02 ± 0.01	0.00 ± 0.00	nd
IL-1α	0.02 ± 0.00	0.13 ± 0.00	nd
IL-1RA	0.11 ± 0.07	0.14 ± 0.06	nd
IL-2	0.11 ± 0.02	0.00 ± 0.00	nd
IL-3	0.06 ± 0.06	0.00 ± 0.00	nd
IL-4	0.04 ± 0.01	0.02 ± 0.01	*
IL-5	0.02 ± 0.00	0.01 ± 0.00	nd
IL-6	0.02 ± 0.00	0.01 ± 0.00	*
IL-8	0.10 ± 0.05	0.07 ± 0.09	*
IL-10	0.16 ± 0.08	0.19 ± 0.05	*
IL-12p40	0.85 ± 0.99	2.39 ± 3.19	nd
IL-12p70	0.27 ± 0.13	0.10 ± 0.00	nd
IL-13	0.95 ± 0.00	0.13 ± 0.00	nd
IL-15	0.53 ± 0.02	0.26 ± 0.06	nd
IL-25	1.48 ± 0.00	0.00 ± 0.00	nd
IL-17F	0.64 ± 0.53	0.45 ± 0.19	nd
IL-18	0.01 ± 0.00	0.00 ± 0.00	nd
IL-22	6.10 ± 0.00	0.00 ± 0.00	nd
IL-27	0.00 ± 0.00	3.78 ± 0.00	nd
M-CSF	26.53 ± 4.13	7.05 ± 1.56	nd
MCP-1	1.01 ± 0.25	0.75 ± 0.15	nd
MCP-3	0.75 ± 0.36	0.00 ± 0.00	nd
MIG	0.07 ± 0.00	0.00 ± 0.00	nd
TGFα	0.36 ± 0.00	0.00 ± 0.00	nd
TNFα	0.22 ± 0.00	0.22 ± 0.00	*
TNFβ	0.71 ± 0.00	0.00 ± 0.00	nd

Results significances are presented through the symbol (*), according to the *p*-value, with one, two, three or four symbols, corresponding to 0.01 < p ≤ 0.05; 0.001 < *p* ≤ 0.01; 0.0001 < *p* ≤ 0.001 and *p* ≤ 0.0001, respectively.

### 3.2 Cytocompatibility assessment

**FIGURE 2:**
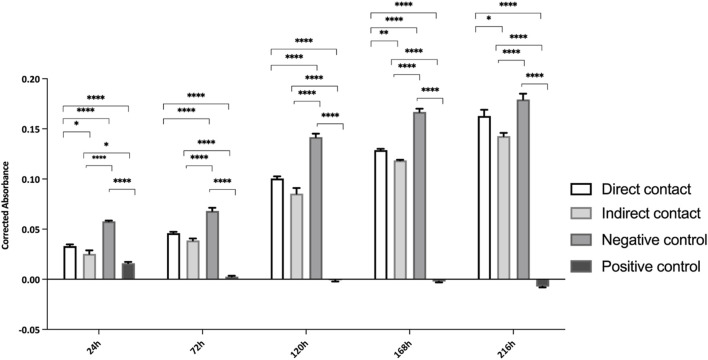
Cytocompatibility tested by PrestoBlue™ viability assay for hDPSCs. Results presented in mean ± SE (standard error of the mean). Results significances are presented through the symbol (*), according to the *p*-value, with one, two, three or four symbols, corresponding to 0.01 < p ≤ 0.05; 0.001 < *p* ≤ 0.01; 0.0001 < *p* ≤ 0.001 and *p* ≤ 0.0001, respectively.

In line with the ISO 10993-5:2009 guidelines, cell viability was evaluated using PrestoBlue™ on various samples of hDPSCs seeded directly in Reaxon^®^ NGCs or through indirect contact. Additionally, control tests without cells were conducted for all groups. The corrected absorbance values for each time point (24, 72, 120, 168 and 216 h) are presented in Figure 2, while Table 3 highlights the statistical differences between the experimental groups at each time point.

**TABLE 3:** Differences were considered statistically significant at *p* ≤ 0.05.

	24 h				72 h				120 h				168 h				216 h			
	A	B	C	D	A	B	C	D	A	B	C	D	A	B	C	D	A	B	C	D
A		*	****	****		ns	****	****		ns	****	****		**	****	****		*	ns	****
B			****	*			****	****			****	****			****	****			****	****
C				****				****				****				****				****
D																				

Results significances are presented through the symbol (*), according to the *p*-value, with one, two, three or four symbols, corresponding to 0.01 < p ≤ 0.05; 0.001 < *p* ≤ 0.01; 0.0001 < *p* ≤ 0.001 and *p* ≤ 0.0001, respectively.

At 24 h, hDPCs cultured in basal medium (negative control) showed a significantly higher viability, with statistically significant differences compared to other groups (p < 0.0001). As expected, the group supplemented with DMSO had the lowest viability at this time point, a fact that continued throughout the assay. The negative control group consistently showed a higher number of cells compared to those seeded in the Reaxon^®^ NGC. However, by the final timepoint (216 h), this difference between the control and the group with cells seeded directly into the Reaxon^®^ NGC was no longer significant (p = 0.0773). Between the two Reaxon^®^ groups, a statistically significant difference (p = 0.0013) appeared at 168 h, demonstrating that the cells seeded directly inside the NGC proliferate more rapidly. Nevertheless, the cells seeded indirectly still maintained good viability, indicating that both the internal and external environments of the NGC are non-cytotoxic and support effective cell adhesion. Although the rate of proliferation is slower compared to the negative control group (cells in medium alone), the Reaxon^®^ NGC remain a suitable environment for cell growth. This indicates that the biomaterial supports cell survival despite a slight initial delay in adhesion and proliferation.

Overall, the negative control group demonstrated high cell viability, with a continuous and steady increase throughout the experiment. In contrast, the test groups showed a noticeable initial delay in metabolic activity, most likely due to the influence of the NGC material. However, this delay was overcome as the viability of the test groups increased significantly. By 168 h, the difference between the groups with Reaxon^®^ NGC and the positive control had diminished, especially in the group where cells were directly seeded into the NGC. This suggests that, despite the slower start, the Reaxon^®^ NGC supports cellular viability and proliferation over time. As expected, the DMSO-supplemented group exhibited the lowest viability from the earliest time points and remained consistently cytotoxic throughout the assay, confirming its detrimental effect on the cells.

**FIGURE 3:**
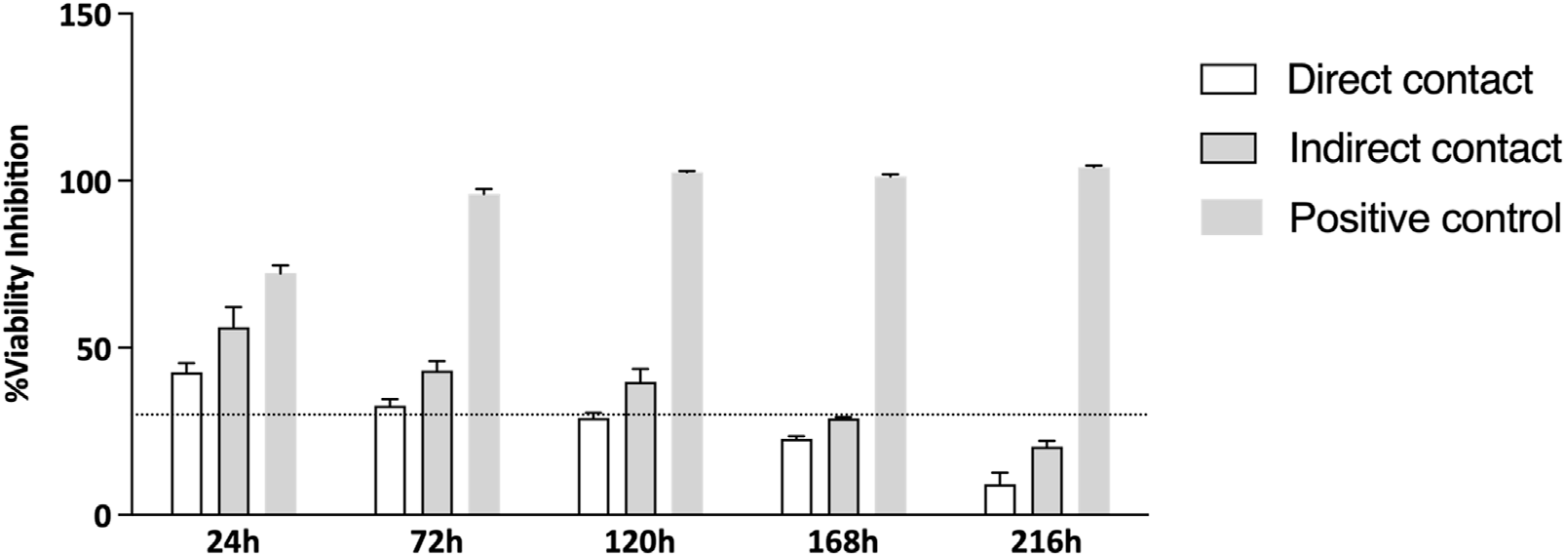
% of viability inhibition assessed by PrestoBlue^®^ viability assay after 24, 72, 120, 168 and 216 h. The results were normalized with the negative control group set at 0%. The dashed line in the graph represents the 30% threshold, which marks the point at which inhibition is considered cytotoxic, as per the ISO 10993-5:2009 guidelines.

Additionally, the percentage of viability inhibition, normalized against the control group, is illustrated in Figure 3. As per Annex 3 of ISO 10993-5:2009, viability inhibition exceeding 30% is classified as cytotoxic, with the threshold shown in Figure 3 by a dashed line. The viability inhibition assay results suggest that the Reaxon^®^ NGC is not cytotoxic. In the first two time points (24 h and 72 h), viability inhibition exceeded 30%, as shown in Figure 3, which indicates a greater challenge for cell growth during this initial phase of adhesion to the NGC. However, at 120 h, the group where cells were directly seeded shows a reduction in viability inhibition, dropping below the cytotoxicity threshold, and continues to decrease significantly until the final time point. Similarly, at 168 h, the group with cells seeded indirectly also experiences a reduction in viability inhibition, which continues until the end of the experiment. The transient delay in adhesion and proliferation observed in this study, as reflected by viability inhibition values above the 30% threshold during the initial stages (up to 168 h) in Figure 3, is consistent with known properties of chitosan-based biomaterials. This phenomenon reflects an adaptation phase where cells adjust to the scaffold surface (Alvites et al., 2021). Importantly, by the study’s endpoint (216 h), viability inhibition for the direct contact group decreased significantly, falling well below the cytotoxic threshold defined by ISO 10993-5:2009. The initial higher inhibition values are attributed to the physicochemical properties of chitosan, such as its hydrophobicity and surface charge, which can transiently limit protein adsorption and cell adhesion. These characteristics are typical of chitosan-based scaffolds and do not indicate inherent cytotoxicity (Bumgardner et al., 2003; Aibani et al., 2021). This behavior highlights the need to consider the dynamic nature of cell-material interactions, particularly for chitosan-based scaffolds. While initial adaptation may occur, the long-term support for cell viability demonstrated in this study affirms the suitability of Reaxon^®^ as a biocompatible material for nerve regeneration applications. Additionally, the use of hDPSC-CM represents a promising strategy to overcome the initial challenges of cell adhesion to chitosan-based scaffolds. CM contains a rich array of bioactive factors that promote nerve regeneration through paracrine effects, bypassing the need for direct cell-material interactions. This approach could enhance the regenerative potential of the scaffold by providing immediate therapeutic benefits while mitigating the adaptation phase observed with live cells. These findings suggest that cells can adhere to the material and proliferate, with the NGCs having no harmful effects. Moreover, the group with direct seeding exhibits faster growth and lower viability inhibition compared to the other group.

### 3.3 Scanning electronic microscopy

**FIGURE 4:**
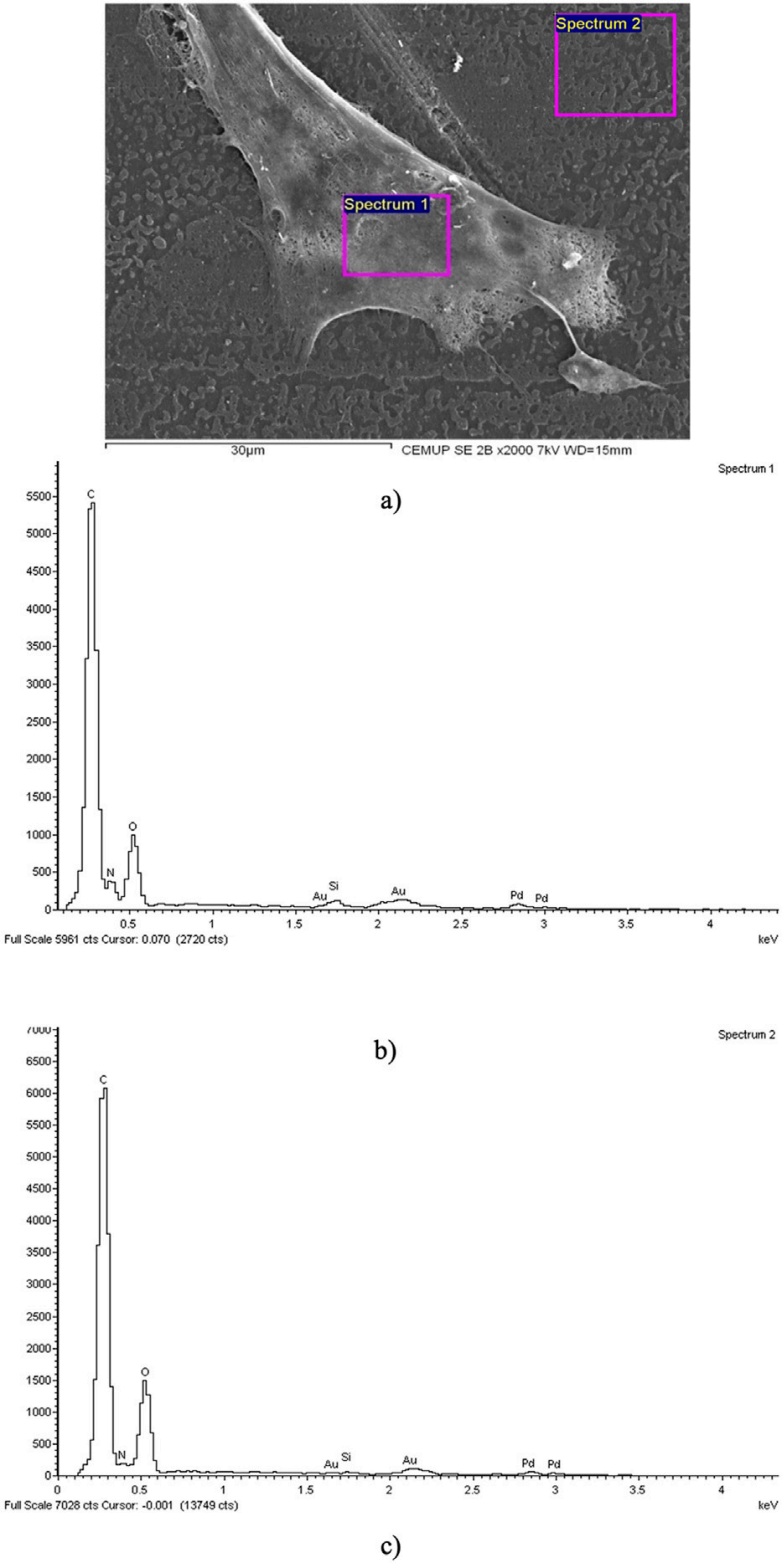
SEM and EDS evaluation of the Reaxon^®^ NGC and hDPSCs: **(A)** hDPSCs cell layer adhered to the inner face of the Reaxon^®^ NGC, magnification: ×2,000; **(B)** EDS evaluation of the Spectrum 1 region hDPSCs; **(C)** EDS evaluation of the Spectrum 2 region-inner surface of the Reaxon^®^ NGC without a cell layer.

After evaluating cytocompatibility and neurogenic differentiation, the samples were prepared for SEM and EDX analysis. The results of this analysis are presented in Figure 4. The findings suggest that hDPSCs adhered and proliferated on the NGC, displaying typical structure and morphology, which highlights the biocompatibility of the material.

The assessment of the samples using SEM provided insights into the inner surface of the Reaxon^®^ NGCs and the morphology of hDPSCs. Additionally, it highlighted the suitability of the biomaterial for cellular adhesion and proliferation, both individually and within homogeneous cell layers (Figure 4A). The EDS analysis further confirmed the presence of cells and uniform cell layers by detecting nitrogen (Figures 4B, C).

### 3.4 Neurogenic differentiation assay

**FIGURE 5:**
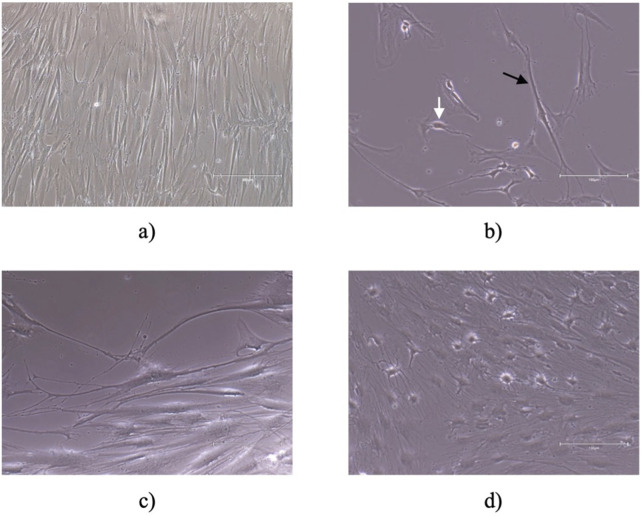
Neurogenic differentiation: **(A)** control; **(B)** 6 h after addition of neurogenic induction medium, cells acquired a neuroglial-like shape with development of axonal-like (black arrow) and dendrite cell structures (white arrow) **(C)** 30 h after addition of neurogenic induction medium and **(D)** 72 h after addition of neurogenic induction medium the cells appear to have developed a network of neural connections. Magnification of **(A)** is ×100 and scale bar 300 μm. Magnification for **(B–D)** is 200 and scale bar 150 μm.

Six hours after the replacement of the culture medium with neurogenic induction medium, hDPSCs exhibited morphological changes indicative of neuronal differentiation. The cells developed extensions resembling dendrites and axons, which are characteristic of neurons. These morphological alterations persisted throughout the 5 days of observation period, becoming more pronounced over time, as shown in Figure 5. However, there was also identified an increase in the mortality rate and number of cell debris in suspension. By 72 h, the cells displayed a network of interconnected structures, suggesting the formation of neural-like connections. These findings underscore the potential of hDPSCs to adopt neuronal characteristics under appropriate induction conditions. In the last years, neuronal differentiation of these cells was demonstrated in several *in vivo* studies using various approaches and methods (Nuti et al., 2016). This study employed morphological changes as a preliminary indicator of neurogenic differentiation potential. While this approach offers an initial understanding, it does not provide definitive evidence of neuronal or glial differentiation. The inclusion of immunocytochemical markers would provide a more comprehensive and detailed evaluation of the differentiation process.

## 4 Discussion

The findings of this study support the development of innovative treatments aimed at addressing the challenges posed by PNI, a major cause of disability that results in motor and sensory deficits, significantly impacting patients’ quality of life. Therefore, PNI continues to be a major clinical problem due its poor capacity for full restoration of function, particularly with long gap nerves. When a traumatic nerve injury occurs, it triggers a process called Wallerian degeneration, which involves the breakdown of the distal axon and synaptic terminals (Dogny et al., 2024; Alvites et al., 2018). This process begins with the accumulation of various compounds around the injury site, leading to disruptions in membrane permeability which triggers an influx of intracellular calcium (Lopes et al., 2022). This influx activates the calpains, proteases capable of cleaving cytoskeletal proteins, such as the neurofilaments and microtubules, resulting in cell damage and destruction (Lopes et al., 2022). Additionally, scar tissue that forms between the proximal and distal ends of a repaired nerve can physically obstruct the axon’s growth (Khodir et al., 2024). Consequently, the target skeletal muscle becomes denervated, leading to muscle atrophy. Over time, the nerve may regenerate and potentially reestablish some functional connections with the muscles. Although peripheral nerves possess an intrinsic ability to regenerate after injury, the healing and regeneration process is often unpredictable and highly dependent on the type and severity of the damage. Although an autologous nerve graft is considered the gold standard for repairing nerve gaps greater than 4 cm, it often leads to donor site complications, limited availability of graft material, and challenges in achieving full functional recovery. For that reason, it is essential to develop new therapeutic interventions that are capable of efficiently promoting nerve regeneration. Stem cell-based treatments alongside with NGCs have become an alternative treatment for improving peripheral nerve regeneration.

NGCs have gained attention as potential replacements for autologous grafts, offering a physical conduit for nerve regeneration while preventing interference from surrounding tissues and scar formation. Nevertheless, the efficacy of NGCs on their own is lower especially in large nerve gaps where the regeneration rate is slower or incomplete. To enhance their effectiveness, NGCs have been combined with MSCs and their CM, which can promote axonal growth and improve clinical outcomes. Specifically, one promising cell type in nerve regeneration strategies is hDPSCs. hDPSCs can differentiate into Schwann cell-like cells under specific conditions, secreting neurotrophic factors that promote axon regeneration and functional recovery. Their minimally invasive extraction from dental pulp makes them uniquely suited for regenerative medicine. The differentiation medium used in this study was designed to promote neurogenic differentiation of hDPSCs. While Schwann cell-like differentiation was not explicitly targeted, the medium’s composition could favor the emergence of such phenotypes. This aligns with prior research showing that hDPSCs can differentiate into Schwann cell-like cells under specific conditions, secreting neurotrophic factors that support axon regeneration and functional recovery. These findings highlight the broad differentiation potential of hDPSCs and their suitability as a model for peripheral nerve regeneration research. Additionally, hDPSCs can release a CM rich in bioactive factors, which may provide a safer and more controlled therapeutic option compared to direct stem cell transplantation. Although the use of CM derived from hDPSCs offers a promising alternative to cell-based therapies, the stability of its bioactive molecules *in vivo* remains a significant consideration. Studies indicate that key neurotrophic factors such as NGF, BDNF, and GDNF exhibit short half-lives, often spanning hours to a few days (Martins et al., 2017; Gonzalez-Gonzalez et al., 2020; Lavorato et al., 2021). However, certain cytokines, including TGF-β and IL-10, show prolonged activity, potentially supporting sustained regenerative responses. The synergistic effect of the complex mixture of factors in CM could compensate for individual limitations in half-life (Gonzalez-Gonzalez et al., 2020). Moreover, strategies for optimizing the delivery of CM, such as loading it directly into chitosan NGCs with Matrigel, could enhance its effectiveness by maintaining localized bioavailability (Luo et al., 2021; Kichenbrand et al., 2019). These considerations highlight the need for further exploration *in vivo* to refine this approach for PNI treatment.

This study evaluated the viability and adhesion of hDPSCs to a commercially available NGC Reaxon^®^, as a preliminary step toward developing a more effective therapy for peripheral nerve regeneration. The focus was ensuring that the cells remained viable in the NGC environment, optimizing conditions for future *in vivo* testing. Also, the hDPSCs-CM at different passages (P4 and P7) was analyzed to determine if there were differences between passages that could be relevant to the *in vivo* applications. CM offers a promising alternative to cell-based therapies, reducing risks like immune rejection, tumorigenicity, and regulatory challenges associated with live cell cultures (Mohd Nor et al., 2023; Kichenbrand et al., 2019). The complex interactions between cytokines, growth factors, and neurotrophic factors involved in peripheral nerve regeneration are still being explored (Yi et al., 2020; Wang et al., 2019). However, the hDPSCs-CM can produce factors that actively contribute to various stages of nerve regeneration, indicating its potential as a therapeutic option. In this analysis, hDPSCs-CM was examined for the presence of interleukins, chemokines, growth factors, immunosuppressive and immunomodulatory factors, and other biomolecules important for enhancing regeneration (Younes et al., 2023; Kichenbrand et al., 2019). Both P4 and P7 passages produced neurotrophic and anti-inflammatory factors, with P4 showing a slightly more pronounced effect in promoting reparative properties. Some biomarkers were present at low concentrations, but effective therapeutic levels of these factors are often found within low yet significant ranges (Gonzalez-Gonzalez et al., 2020). P4 seemed to prioritize anti-inflammatory responses, indicated by the presence of IL-4 and IL-5, and demonstrated immune-modulatory functions through IL-2, IL-4, and IL-5, which could be beneficial in the early stages of healing (Junttila, 2018). P7 exhibited enhanced immune recruitment capabilities, as evidenced by higher levels of MCP-1 (Caseiro et al., 2019; Liu et al., 2023). Both passages contained TNF-α, which helps recruit immune cells like T cells, monocytes, and neutrophils to clear infections and debris; this cytokine was more abundant in P7 (de Gonzalo-Calvo et al., 2010). However, G-CSF was only present in P7, contributing to immune cell recruitment as well. Eotaxin was also present, supporting immune cell migration and facilitating the resolution of inflammation and tissue repair, present in both passages but higher in P7 (Nelson et al., 2024). In terms of modulating early immune responses, IFN-γ, IL-1α, and IL-12p70 are crucial for infection control, while IL-13 and IL-18, found at higher levels in P4, assist in tissue repair and maintaining an anti-inflammatory environment (Salkin et al., 2023; Llibre and Duffy, 2018). IL-15, present in both passages, indicates stronger activation of T cells and NK cells, promoting robust immune responses that are beneficial for fighting infections or tumors. P4 displayed higher levels of IL-25, which stimulates Th2-type immune responses involved in anti-inflammatory actions and allergic reactions (Kokubo et al., 2022). IL-6, a key pro-inflammatory cytokine, was also higher in P4, indicating active immune responses that aid in defending against infections. IL-27 plays a role in regulating immune responses, while IL-17F is involved in inflammation. TGF-α is a growth factor that promotes cell proliferation, plays a vital role in tissue repair and regeneration, and can influence immune responses (Ghasemi et al., 2023). In summary, TNF-α is more associated with inflammation and immune activation, with higher levels observed in P7, while TNF-β plays a regulatory role in lymphocyte function and immune homeostasis, being present only in P4 (Kokubo et al., 2022). Overall, P4 higher levels of key cytokines suggest a broader and more intense biological activity, which could be more favorable in situations requiring robust immune responses and tissue regeneration. In contrast, P7 profile, though showing fewer biomolecules overall, may be more targeted for specific functions like chemotaxis, providing more controlled responses in certain contexts. The analysis revealed that P4 exhibited lower levels of IL-10, a key anti-inflammatory cytokine, compared to P7. IL-10 plays an important role in reducing chronic inflammation and supporting tissue repair. The lower IL-10 levels in P4 suggest a CM profile focused on early-stage repair, characterized by elevated IL-15, IL-25, MCP-3, and IL-6, which promote immune recruitment and tissue remodeling. This aligns with the initial phases of peripheral nerve regeneration. Conversely, higher IL-10 levels in P7 indicate a shift towards inflammation resolution and long-term recovery, making it more suitable for sustained anti-inflammatory effects. These findings highlight the need to tailor CM use according to the therapeutic phase, with P4 optimizing initial repair and P7 enhancing recovery. The temporal evolution of cytokine profiles reflects hDPSCs’ ability to adapt their CM for specific stages of regeneration. Future studies should explore these variations *in vivo* to confirm their implications for peripheral nerve repair.

Following hDPSCs-CM analyses, the cytocompatibility and neurogenic differentiation abilities were assessed using a set of well-established methodologies. The assessment of neurogenic differentiation in this study was based on morphological changes as an initial approach. While these findings provide preliminary evidence of differentiation potential, future analyses using immunocytochemical markers will be essential to confirm and quantify neuronal and glial differentiation. The cytocompatibility characteristics of all the test groups between hDPSCs and Reaxon are non-cytotoxic in respect to cell viability and proliferation according to ISO 10993-5:2009 standards. Metabolic data from PrestoBlue™ analysis revealed that the Reaxon^®^ NGC induce an increase in cell viability both in the groups were seeded directly and indirectly. The assay results indicated that chitosan conduits initially slowed down cell adhesion, with differences in viability and metabolic activity noted until the 48-h time point when compared to the hDPSCs cultured only in the standard medium. However, after this time point, the viability associated to the biomaterial began to improve. The group of cells seeded directly reached the viability level of the negative control group (DMEM 10%) more closely over time, and at the last time point, there were no statistically significant differences between both groups. In contrast, the group in which the cells were seeded indirectly showed slightly lower viability values. Our group had already studied the cytocompatibility of Reaxon^®^ with other type of MSCs (Alvites et al., 2021). The results showed that the NGC not only supported the adhesion and proliferation of olfactory mucosa mesenchymal stem/stromal cells but also exhibited no toxicity. Viability assay results showed that the cells cultured in chitosan maintained high viability levels, comparable to the negative control group (DMEM 10%). This confirmation of cytocompatibility is essential to ensure that the combination of chitosan and MSCs promotes a favorable environment for nerve regeneration (Alvites et al., 2021). Our results are in accordance with the findings from this study, reinforcing the effectiveness of chitosan as a scaffold material in supporting the viability and proliferation of MSCs, in this case hDPSCs to promote nerve regeneration.

The initial interaction between hDPSCs and chitosan NGCs is key to creating a regenerative microenvironment, as early delays in adhesion may reflect cellular adaptation to the biomaterial. This adaptation phase could transiently enhance the secretion of pro-inflammatory cytokines, survival factors, or matrix remodeling enzymes, which serve to mediate cellular attachment to the biomaterial. As the cells stabilize and proliferate on the chitosan scaffold, the CM is likely to shift toward the production of growth-promoting and neurotrophic factors, as seen in other studies involving MSCs and biomaterials. These changes in the composition of the CM could be critical for enhancing its therapeutic potential, particularly in promoting axonal regeneration and reducing inflammation. Furthermore, the interplay between cell behavior and scaffold properties could influence not only the concentration of secreted factors but also their bioactivity. The delayed adhesion might reduce the immediate availability of certain factors crucial for early nerve regeneration, while the sustained presence of viable cells on the scaffold could ensure a prolonged release of key biomolecules over time (Yi et al., 2020; Ghasemi et al., 2023). This dynamic could be particularly advantageous in *in vivo* applications, where a continuous supply of growth factors and cytokines is necessary to support the sequential stages of nerve repair. These findings emphasize the importance of considering the temporal evolution of the CM composition in response to cell-material interactions. Moreover, exploring how these early cellular dynamics influence the functional efficacy of CM in *in vivo* models could further validate the translational potential of this approach in peripheral nerve regeneration.”

To confirm the interaction between hDPSCs and Reaxon^®^ conduits, the chitosan NGCs where the cells were seeded were analyzed using SEM and EDS. The results indicate that the NGC composition creates a conducive microenvironment for the attachment and growth of hDPSCs. These findings align with the cytocompatibility test results, which showed significant cell proliferation and high viability. The observed increase in cell proliferation suggests that the NGC offer a supportive environment for cellular growth, which is crucial for nerve regeneration. Using EDS in combination with SEM allows for the identification of elements present near the surface of the sample, creating a graphic map of the chemical components detected through emitted X-ray energy. In this situation, the analysis revealed a predominance of carbon and oxygen, which is consistent with the chemical composition of chitosan. Additionally, the evaluation of the regions of **Spectrum 1** and **Spectrum 2** showed nitrogen peaks, indicating the presence of cellular material adhering to the surfaces of the NGC. This confirms that the structures identified in the SEM images correspond to hDPSCs attached to the internal surface of the chitosan NGC.

The study focused primarily on the *in vitro* viability and adhesion of hDPSCs in NGCs, the next step will involve translating these findings into *in vivo* models of PNI, such as sciatic nerve defects in rats. The rat sciatic nerve model is widely used in nerve regeneration research due to its anatomical similarities to human peripheral nerves and the availability of standardized assessment methods for functional recovery. However, it is important to note that rodents have faster nerve regeneration rates compared to humans, which may limit the direct applicability of the findings. Larger animal models, such as ovine, with nerve dimensions more comparable to human nerves, would provide a more accurate representation of the clinical scenario and will be considered for further research, if the rat model provides promising results. Ultimately, the goal of combining hDPSCs with NGCs is to develop a synergistic approach that addresses the limitations of each individual component. The NGC provides the necessary structural support and protection for regenerating axons, while hDPSCs offer the biological stimuli required for effective nerve regeneration. This combined approach has the potential to offer a viable alternative to autologous nerve grafting, particularly for large nerve gaps where traditional methods are less effective.

## 5 Conclusion

The combination of MSCs and biomaterials to support peripheral nerve regeneration after PNI is an increasingly adopted therapeutic approach that has shown promising results. The hDPSCs seem to have desirable traits for nerve regeneration, and the application of chitosan NGCs has demonstrated effectiveness in this context. Preliminary biological characterization of hDPSCs demonstrated a propensity for neurogenic differentiation. Additionally, cytocompatibility assessments indicated an absence of cytotoxic effects. These findings further support the safety of using these cells in therapeutic applications.

In conclusion, the findings from this study provide a robust basis for advancing hDPSC-based therapeutic strategies in peripheral nerve regeneration. By refining the conditions that promote cell viability and adhesion within NGCs, and further investigating the bioactive components of hDPSC-CM, significant strides can be made toward developing more effective and reliable treatments for PNI. These advancements could potentially lead to improved clinical outcomes in the context of PNI treatment.

## Data Availability

The original contributions presented in the study are included in the article/supplementary material, further inquiries can be directed to the corresponding author.

## Abbreviations

CM, Conditioned Medium; DMSO, Dimethyl Sulfoxide; DPBS, Dulbecco’s Phosphate-Buffered Saline; DPSCs, Dental Pulp Stem Cells; EDS, Energy Dispersive X-ray Spectroscopy; EGF, Epidermal Growth Factor Recombinant Protein; FBS, Fetal Bovine Serum; G-CSF, Granulocyte Colony-Stimulating Factor; GM-CSF, Granulocyte-Macrophage Colony-Stimulating Factor; GROα, Growth Regulated Oncogene Alpha; hDPSCs, Human Dental Pulp Stem Cells; hDPSCs-CM, Conditioned medium from hDPSCs; HMDS, Hexamethyldisilazane; IFNγ, Interferon Gamma; IL, Interleukin; IP-10, Interferon-Gamma Inducible Protein 10; MCP-1, Monocyte Chemoattractant Protein-1; MDC, Macrophage Derived Chemokine; MIP, Macrophage Inflammatory Protein; MSCs, Mesenchymal Stem Cells; NGCs, Nerve Guide Conduits; PDGF-AA, Platelet-Derived Growth Factor-AA; PNI, Peripheral Nerve Injury; RANTES, Regulated on Activation, Normal T Cell Expressed and Secreted; SEM, Standard Error of the Mean; TGFα, Transforming Growth Factor Alpha; TGFβ, Transforming Growth Factor Beta; TNFα, Tumor Necrosis Factor Alpha; VEGF-A, Vascular Endothelial Growth Factor A.
